# Higher education management in western regions by educational power strategy and positive psychology

**DOI:** 10.3389/fpsyg.2023.1015759

**Published:** 2023-02-16

**Authors:** Xiaomeng Sun

**Affiliations:** Normal College, Shihezi University, Shihezi, China

**Keywords:** strategy of strengthening the country through education, positive psychology, western region, higher education, educational resources

## Abstract

With the deepening of the strategy of strengthening the country through education, the innovation and development of higher education, system reform and teaching innovation in the western region have become the focus of researchers’ attention, and the optimization of educational power strategy has always been an important basis for the development of teaching work. On the basis of fuzzy models Takagi and Sugeno (T–S), this paper constructs an educational resource recommendation model based on T–S fuzzy neural network, verifies the feasibility of the model, further combines the educational resource recommendation model with university teaching, and analyzes the application effect. The current situation of educational resources investigation in M College is analyzed. It is found that the full-time teachers’ overall academic qualifications are not high, the proportion of young full-time teachers with certain experience is small, and the professional advantages of the school are not obvious. After applying the educational resource recommendation model, the accuracy of educational resource recommendation is obviously improved, and the design is feasible. The educational management mode with positive psychological emotions has a good teaching effect, which can greatly improve teachers’ dedication and concentration. Positive psychological emotions can reduce the possibility of intensification of contradictions and the possibility of behavioral opposition. Teaching resource recommendation mode can improve college students’ interest in the application of teaching resources to a certain extent, and their application satisfaction is obviously improved. This paper not only provides technical support for the improvement of teaching management resource recommendation mode, but also contributes to the optimization of teaching power strategy.

## 1. Introduction

With the continuous strengthening of China’s economic strength, building an educational power has become a necessary task for China to promote the development of higher education (Dopson et al., 2019). In particular, it plays an important role in the construction of higher education in western China. Due to the limitations of various conditions, the overall level of education in western colleges and universities has no advantages compared with that in the eastern region. Strengthening the construction of higher education in the western region has become an important construction task for implementing the strategy of strengthening the country through education.

In the context of continuous social development, higher education has become an important cornerstone of current social development. College and university education are not only important sources of high-quality talent output but also an important base for scientific and technological innovation. Therefore, strengthening the innovation and development of college and university education is one of the main tasks of current society. However, at present, many colleges still do not pay enough attention to education reform under the premise of building a strong educational country. The traditional school education development model has greatly reduced the quality of education, and it is difficult to achieve the construction of a strong education country (Runhong, 2022). In order to implement the strategy of strengthening the country through education, colleges and universities need to recognize the fundamental tasks of education, innovate training objectives, implement teaching reform, optimize curriculum institutions, improve the level of teaching activities, and strengthen the construction of teaching staff. The construction of higher education in the western region plays an important role in the construction of a powerful country in higher education due to its special orientation (Dong et al., 2019). In recent years, due to the constraints of geographical location, economic development, teaching scientific settings, education mode, and other factors, China’s higher education has shown a pattern of “the east region is strong and the west region is weak.” It causes a large academic brain drain in the western region, the rigid personnel management system, weak social service ability, and other issues (Fu et al., 2021). Topics such as innovation and the development of higher education in the western region, system reform, teaching innovation, and entrepreneurship education have become the focus and research objects of all walks of life. Carlucci et al. (2018) proposed a framework in the study to analyze students’ evaluations of the quality of higher education teaching and to reveal the risks that affect the quality of teaching and the quality of courses requiring continuous improvement. The framework integrates two decision-based approaches: a standardized U control chart and ABC analysis using fuzzy weights. Using student ratings, control charts can identify courses that need to improve teaching quality in the short term. ABC analysis uses fuzzy weights to deal with the fuzziness and uncertainty of students’ teaching evaluations and provides a risk map of potential areas for long-term teaching performance improvement. The proposed framework allows for the prioritization of corrective measures needed to respond to student criticism of teaching and curriculum quality (Carlucci et al., 2018). Li et al. (2020) used the comparative analysis method to conduct a vertical evolution analysis and a horizontal comparative analysis of the level of higher education investment in the western region and the status quo of regional economic development (Li et al., 2020). Shen and Ho (2020) proposed a hybrid bibliometric method combining direct citation network analysis and text analysis to visually examine papers on higher education retrieved from the scientific network database (Shen and Ho, 2020). Sun (2020) analyzed the importance of psychological quality in college students’ entrepreneurial education from the perspective of positive psychology and explored the educational mode of combining network information technology with maker education to cultivate college students’ innovation and entrepreneurship ability (Sun, 2020). Godoy-Bejarano et al. (2020) provided evidence of how higher environmental complexity alters the slack-performance relationship in the long run by introducing three effects: efficiency effects, profitability effects, and incentive effects. After measuring these effects on the Colombian team, the results showed that the company had implemented a variety of simultaneous and purposeful actions against organizational slack to compete in a more complex environment (Godoy-Bejarano et al., 2020). Garland (2021) pointed out that positive psychology paid more attention to the positive qualities of human beings. The function of positive emotions is to expand and construct direct thoughts or behaviors of individuals, to provide sufficient resources for direct thoughts or behaviors of individuals, and to enable individuals to respond more accurately, perceive more comprehensively, and think more creatively (Garland, 2021). According to Guo et al. (2020), it is critical to implement positive psychology-based innovation and reform in college education. Network education resources play an important role in college education and are the most valuable form of teaching. However, the update speed of network resources is fast, and the utilization efficiency is low. Educational resources play an important role in schools, and educational resource recommendation plays a crucial mediating role in student learning (Guo et al., 2020). De Medio et al. (2020) proposed a fuzzy predictive control algorithm based on the Takagi and Sugeno (T–S) fuzzy model and integrated the nonlinear model into the neural network model to solve the problem of an insufficient linear relationship in the educational resource recommendation algorithm. It can be used to design educational resource recommendations (De Medio et al., 2020). Matos Pedro et al. (2020) indicated that intellectual capital in higher education institutions had a positive impact on institutional performance through relational capital and structural capital. Meanwhile, the quality of life has become an important aspect of performance standards in higher education institutions, especially in terms of students’ perceptions of the quality of academic life. When higher education institutions understand and measure their intellectual capital, they will better understand their core competencies. This enables better allocation of resources and more effective strategic and operational actions (Matos Pedro et al., 2020). Li et al. (2022) focused on the use of big data and mobile computing-driven models to evaluate classroom teaching performance in their research. In addition, in the era of educational big data, their research also explored the general process of teachers’ acquisition, analysis, and use of educational data to improve teaching performance. The data mining method and mobile data collection were organically combined into the benchmarking analysis. The classroom teaching performance of local colleges and universities was evaluated to enrich the teaching management theories and methods of local colleges and universities. The research results showed that benchmarking analysis could produce more meaningful results, which provided new data support for improving the quality of teaching management (Li et al., 2022).

Under the background of the transformation of China’s economic development mode and the deepening of education and teaching reform, and under the background of the strategic development of a powerful country in education, this study combines the theory of positive emotions in positive psychology and the fuzzy predictive control algorithm to construct the T–S fuzzy neural network educational resource recommendation model. The application effect of the model is studied and analyzed. The innovation lies in the strategic level of strengthening the country through education, using the relevant theories of positive psychology, innovating in the way of recommending educational resources in resource colleges and universities, and constructing a teaching resource recommendation model for higher education management in the western region. The current situation of teaching and the feasibility and effect of model application are investigated and analyzed. The purpose is to improve the teaching management level of college students, improve the utilization rate and satisfaction of teaching resources, make teaching resources more widely used, and increase their utility.

## 2. Theoretical research and model method design

### 2.1. The strategic thought of education power and the theory of positive psychology

#### 2.1.1. The influence of the strategic thought of powerful education on college education management

**Figure 1:**
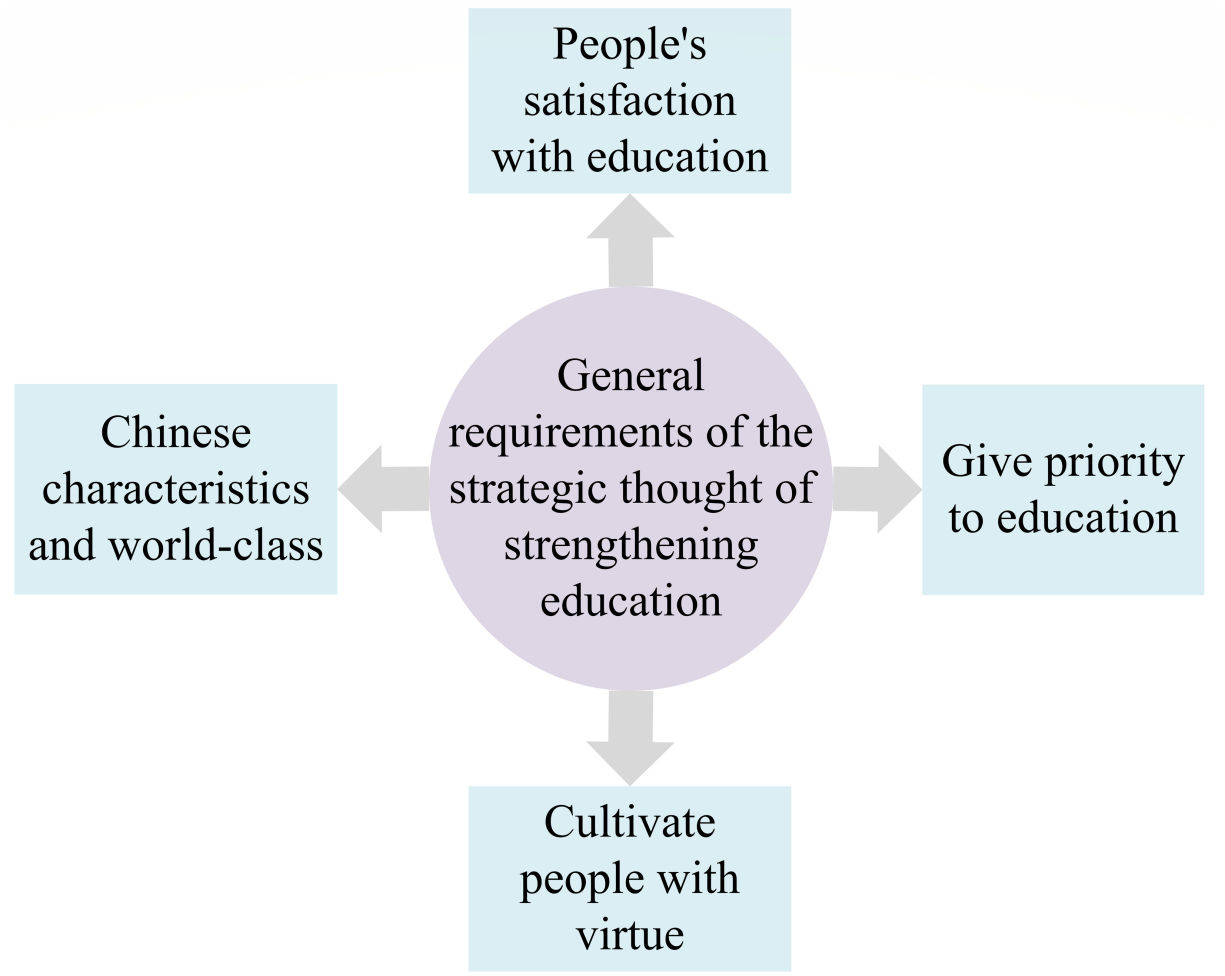
Reflection of the general requirements of the strategic thinking of strengthening the country through education.

Building a strong country in education is the basic project for the great rejuvenation of the Chinese nation (Carr et al., 2021). General Secretary Xi Jinping has drawn a grand blueprint for the strategy of strengthening the country through education, planned a clear path, and provided scientific guidance and actions to follow. A strong country in education depends on whether the comprehensive strength, training ability, international competitiveness, and influence of education have a prominent position (Taufik, 2020). Adhering to education reform and innovation is the fundamental driving force for realizing the strategy of strengthening the country through education. If the construction of an educational power is not good, there will be insufficient intellectual resources to support international technological innovation and technological competitiveness. The essence of building a strong country in education is to improve the quality of education and promote equity in education (Li and Deng, 2022). The overall requirements of the new era for the strategic thinking of strengthening the country through education are reflected in four aspects, as shown in Figure 1.

First, people’s satisfaction with education is the valuable goal of building an educational power. Second, giving priority to education is the basic requirement for building a strong educational country. Third, building morality and cultivating people is the fundamental task of building a powerful country through education (Renigier-Biłozor et al., 2019). Fourth, Chinese characteristics and world-class education are the scientific orientation for developing a powerful country. Based on the strategic thinking of strengthening the country through education, and under the impetus of the new round of the western development policy, the state has provided “blood-transfusion” support for higher education in the western region, continuously supplemented the shortcomings in human, financial, and material resources, and continuously built an industrial service chain connecting the inside and outside of the western region to enhance the conversion rate of scientific research achievements in colleges and universities (Dana et al., 2021).

As an important support for the construction of a strong country, higher education is a key area for the development of the western region and an overall boost to the economy and society in the western region. Integrating educational resources on the basis of the industrial service chain can not only improve the effect of collaborative education, but also provide development opportunities for forming the characteristics of western universities and adjusting the local industrial structure. Under the strategic thinking of strengthening the country through education, the western region should coordinate and integrate innovation, system innovation, and institutional innovation to realize fundamental changes in the way resources are acquired and the transformation of scientific research results.

#### 2.1.2. The effect of positive psychology theory on educational management In colleges and universities

Positive psychology is to use the existing, relatively complete psychological research methods to explore the development of human potential, the satisfaction of needs and the improvement of psychological quality to finally obtain a happy life. Positive psychology emphasizes putting people first and advocates human care for all students. Its purpose is to stimulate and cultivate students’ individual constructive strength and positive psychological quality, guide students to give full play to their self-help and self-healing ability when they encounter problems and difficulties, and finally realize students’ self-development (Baig et al., 2021). Secondly, positive psychology attaches great importance to prevention and promotion of growth, and advocates that, in the face of complex student work problems, individuals should be guided to use their own positive qualities and strengths to stop the problems in their infancy. Positive psychology enables students to understand their own strengths and weaknesses more comprehensively and accurately, and rebuild self-confidence (Kim et al., 2021).

**Figure 2:**
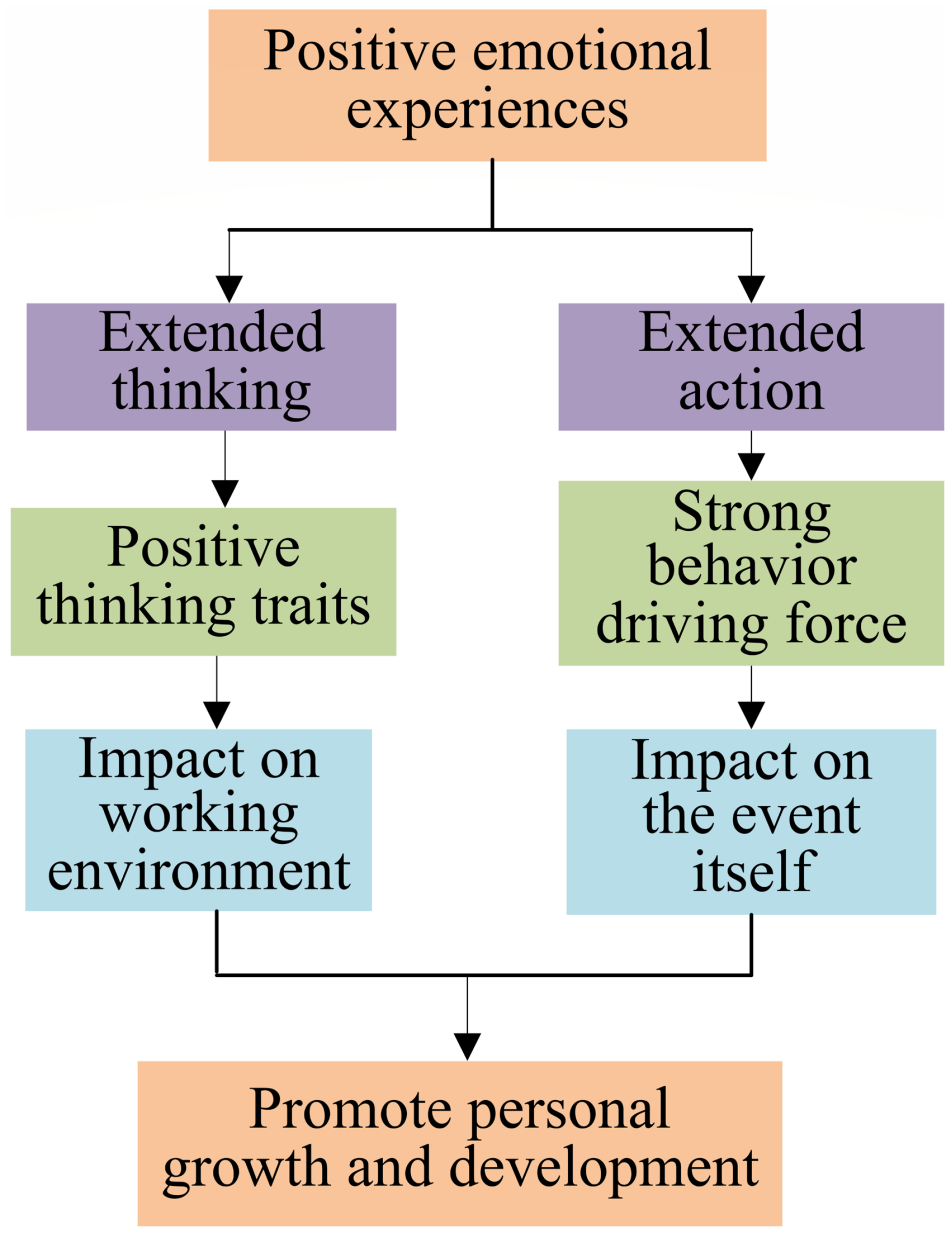
An extended theory of positive emotions.

Positive emotional experience is a branch of research in positive psychology. The function of positive emotions is the ability to expand and structure a person’s direct thoughts or behaviors. Positive emotions provide sufficient resources for an individual’s direct thoughts or actions, enabling individuals to respond more accurately, to be more comprehensive, and to think creatively. In addition to schools and families, the cultivation of college students’ self-management ability is more important than the role of their own psychology (Wang et al., 2022). Therefore, positive psychology plays an important role in higher education management and student teaching. Positive emotional experiences can not only reflect personal happiness, but also benefit personal growth and development. Positive emotions can expand students’ thinking and action (Rodríguez-Campo et al., 2022). The specific extension construction is shown in Figure 2.

In Figure 2, positive emotional experiences provide an opportunity to build sustainable personal resources. Positive emotional experience shows people’s positive thinking characteristics and strong behavioral drive. This kind of emotion affects people’s work attitudes and events and then influences the work environment and the results of work events. Positive emotions provide the potential for personal growth and development.

### 2.2. Construction of higher education management resource recommendation model based on T–S fuzzy neural network

#### 2.2.1. Relationship between recommendation mode of educational management resources and positive psychological emotion and design of fuzzy control algorithm

##### 2.2.1.1. The relationship between the recommended mode of educational resource management and positive psychological emotion

The first priority in the advancement of teaching work in colleges and universities is the optimization of teaching power strategy, the most important of which is the acquisition and application of educational management resources. The recommendation system for educational management resources in colleges and universities includes modules such as acquisition of educational resources, educational resources, recommendation resources, personal educational resources, and subject information management. Among them, the manner in which educational resources are obtained has changed dramatically in recent years. From the traditional book acquisition method to the current information acquisition method, the great change in the method of obtaining educational resources has also had a great impact on educational enthusiasm. Educational resources are also called “educational economic conditions.” The educational process occupies, uses, and consumes human, material, and financial resources, that is, the sum of educational human resources, material resources, and financial resources. Human resources include educators’ and educators’ human resources, that is, the number of students in a school, class, enrollment, graduates, administrators, teaching staff, teaching assistants, workers, production staff, etc. Material resources include fixed assets, materials, and low-value consumables in schools. Fixed assets are divided into common fixed assets, fixed assets for teaching and scientific research, and other general equipment fixed assets. Recommending resources refers to the formation of an educational resource recommendation system through information technology, so as to improve the acquisition effect of teaching resources. Specialty and skill resources, professional resources, personal contacts resources, regional resources, and so on are all examples of personal educational resources (Xiong et al., 2021). Discipline management entails implementing discipline management using information technology and improving the effectiveness of discipline management.

**Figure 3:**
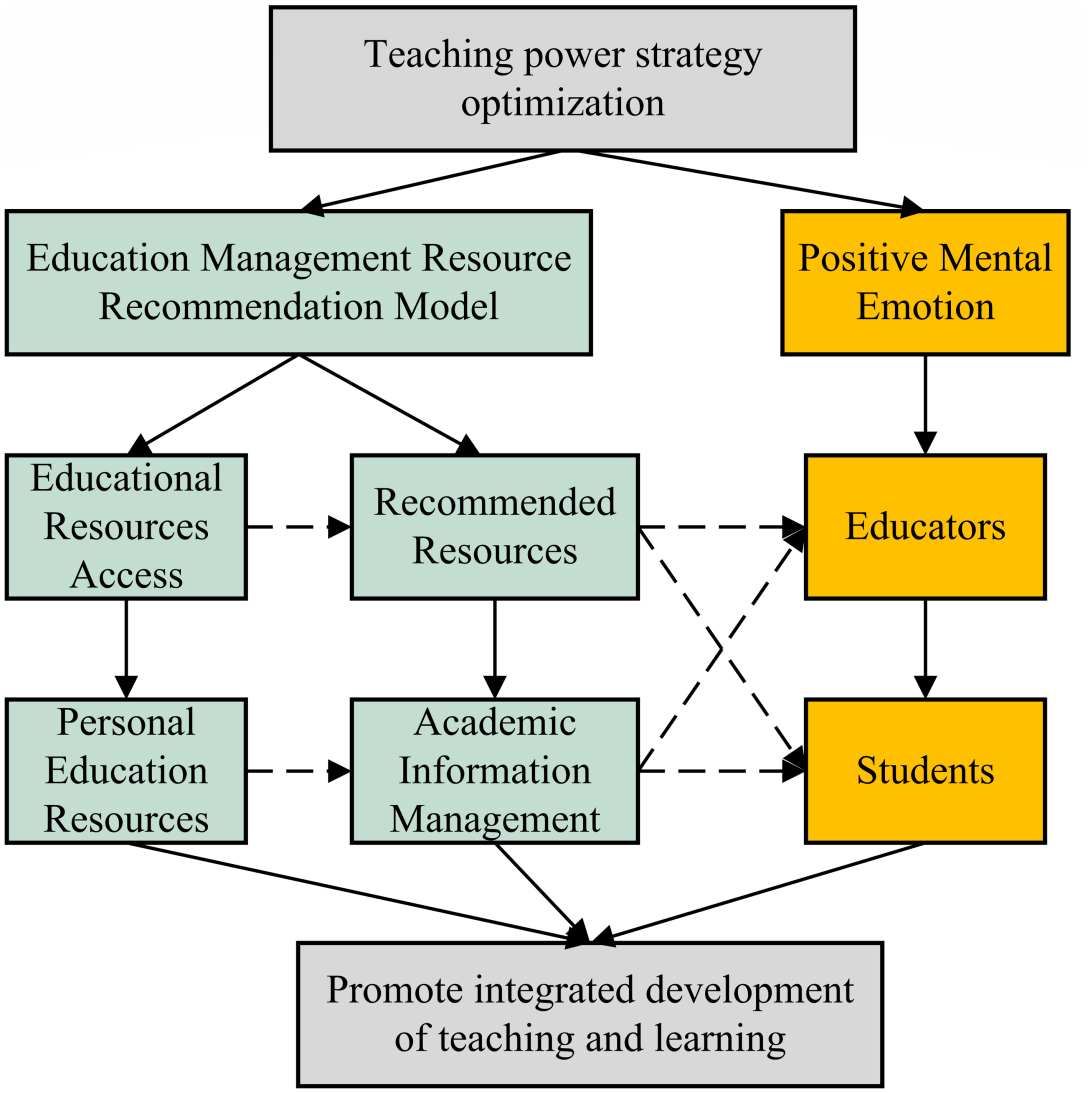
Design of research ideas.

To sum up, the current recommended mode of educational resource management is basically realized by information technology, so it can improve the effect of educational resource management, thereby helping teachers and learners to improve their enthusiasm. Meanwhile, constantly updated educational resource management methods and contents can effectively stimulate educators’ and learners’ enthusiasm in teaching and learning, thereby comprehensively promoting the development of teaching work. Based on this, this paper combines the recommended mode of educational resources management with positive psychological and emotional factors to study the teaching work in colleges and universities to promote the development of teaching work. Figure 3 shows the basic idea of this research.

In Figure 3, under the background of the optimization of teaching power strategy, the optimization of teaching resource management methods and the promotion of enthusiasm can effectively promote the development of teaching work, thus enhancing the development effect of current colleges and universities.

##### 2.2.1.2. Fuzzy control algorithm design

The fuzzy control algorithm is essentially a kind of computer numerical control. It consists of fuzzy set theory, fuzzy linguistic variables, and fuzzy logic reasoning (Kong et al., 2019). The fuzzy predictive control algorithm combines the advantages of the fuzzy and the predictive control algorithm and combines the fuzzy ideas and prediction methods of the two. The effect of predictive control is enhanced through fuzzy prediction, feedback correction, and rolling optimization (Thamallah et al., 2019). Among the forms of fuzzy sets, there are three common ones. The first is the Zadeh form (Lin et al., 2021). The fuzzy set A is shown in Eq. 1:


(1) $$A=\frac{\mu_{A}\left(x_{1}\right)}{x_{1}}+\frac{\mu_{A}\left(x_{2}\right)}{x_{2}}+\cdots +\frac{\mu_{A}\left(x_{n}\right)}{x_{n}}$$

$\frac{\mu_{A}\left(x_{1}\right)}{x_{1}}$ represents the correspondence between the element $x_{1}$ andthe degree of membership $\mu_{A}\left(x_{1}\right)$ . In ordinal even form, the expression of fuzzy set A is shown in Eq. 2:


(2) $$A=\left\{\left(x_{1},\;\mu_{A}\left(x_{1}\right)\right),\left(x_{2},\;\mu_{A}\left(x_{2}\right)\right),\cdots ,\left(x_{n},\;\mu_{A}\left(x_{n}\right)\right)|x\in U\right\}$$

In vector form, the fuzzy set A is given by Eq. 3:


(3) $$A=\left[\mu_{A}\left(x_{1}\right)\mu_{A}\left(x_{2}\right)\cdots \mu_{A}\left(x_{n}\right)\right]$$

In Eqs 1–3, when the degree of membership is 0, this term is omitted in the Zadeh form and the ordinal pair form. In vector form, this item cannot be omitted. In practice, membership function is a key part of fuzzy set theory, which quantitatively describes fuzzy concepts. The general fuzzy distribution functions are divided into normality, upper and lower ring types (Anil Naik, 2022).

#### 2.2.2. T–S fuzzy model rule design

Takagi and Sugeno (T–S) was created by Takagi and Sugeno, referred to as the T–S fuzzy model. The essence is to divide the input space into multiple fuzzy subspaces and build a linear relationship model between input and output in the subspace, following the If-then rule (Zeng et al., 2019).

In the fuzzy model, the input vector x is shown in Eq. 4:


(4) $$x={\left[x_{1},\;x_{2},\cdots x_{i},\;x_{n}\right]}^{T}$$

Among them, $x_{i}$ is a fuzzy language variable. The expression of $T\left(x_{i}\right)$ is shown in Eq. 5:


(5) $$T\left(x_{i}\right)=\left\{A_{i}^{1},\;A_{i}^{2},\cdots ,\;A_{i}^{m_{i}}\right\}\left(i=1,2,\cdots ,\;n\right)$$

$A_{i}^{j}\left(j=1,2,\cdots ,\;m_{i}\right)$ represents the *j*-th language variable value of $x_{i}\text{,}$ representing a fuzzy set of $U_{i}$ . The expression of the membership function is shown in Eq. 6:


(6) $$\mu_{A_{i}^{\prime }}\left(x_{i}\right)\left(i=1,2,\cdots ,\;m_{i}\right)$$

T–S Fuzzy Model Rule Consequences represent linear combinations of input variables. Suppose $x_{1}$ is $A_{1}^{j}\text{,}$ $x_{2}$ is $A_{2}^{j}\text{.}$ Then, $x_{n}$ is $A_{n}^{j}$ . The expression of $y_{j}$ is shown in Eq. 7:


(7) $$y_{j}=p_{j0}+p_{j1}x_{1}+\cdots p_{jn}x_{n}$$

Among them, j=1,2,⋯,m, $m\leq \overset{n}{\underset{i=1}{\prod }}m_{i}$ .

If the input quantity uses the fuzzification method of a single-point fuzzy set, for the given input x, the rule fitness $\alpha_{j}$ is obtained, as shown in Eq. 8:


(8) $$\alpha_{j}=\mu_{A_{1}^{j}}\left(x_{1}\right)\wedge \mu_{A_{2}^{j}}\left(x_{2}\right)\cdots \wedge \mu_{A_{n}^{j}}\left(x_{n}\right)$$

The output of the fuzzy system is the weighted average of the output of each rule, represented by y, as shown in Eq. 9:


(9) $$y=\frac{\sum_{j=1}^{m}\alpha_{j}y_{j}}{\sum_{j=1}^{m}\alpha_{j}}=\overset{m}{\underset{j=1}{\sum }}{\bar{\alpha }}_{j}y_{j}$$

${\bar{\alpha }}_{j}$ is shown in Eq. 10:


(10) $${\bar{\alpha }}_{j}=\alpha_{j}/\overset{m}{\underset{i=1}{\sum }}\alpha_{i}$$

Suppose: there are m rules. Then, the *i*-th rule is shown in Eq. 11:


(11) $$\begin{array}{l} R_{i}:\mathrm{If}\;x\left(t\right)\;\mathrm{is}\;A_{i}\;\mathrm{then}\;y_{i}\left(t+1\right)=a_{i0}+a_{i1}x_{t1}\\ +\cdots +a_{im}x_{tm}i=1,2,\cdots ,m \end{array}$$

In fuzzy rules, $x\left(t\right)$ is shown in Eq. 12:


(12) $$x\left(t\right)=\left[x_{t1},\;x_{t2},\cdots x_{ts}\right]$$

In the If-then rule, the part before the then statement is called the antecedent; $A_{i}$ is a fuzzy set composed of regression vectors; the part after the then statement is called If. Then rule consequences represent linear combinations of input and output data. In the If-then rule, the data information of the rule’s consequent can only be obtained if the antecedents of the then rule are satisfied (Yi et al., 2021).

The least-squares method is used to resolve the regular consequent to obtain $a_{im}\text{.}$ If the membership degree of $x\left(t\right)$ to the *i*-th rule is $\mu_{i}\left(t\right)\text{,}$ the output is shown in Eq. 13:


(13) $$\hat{y}\left(t\right)=\overset{m}{\underset{i=1}{\sum }}\beta_{i}\left(t\right)y_{i}\left(t\right)$$

$\beta_{i}\left(t\right)$ is shown in Eq. 14:


(14) $$\beta_{i}\left(t\right)=\mu_{i}\left(t\right)/\overset{m}{\underset{i=1}{\sum }}\mu_{i}\left(t\right)$$

#### 2.2.3. Design of recommendation process for higher education management resources based on T–S fuzzy neural network

Previously, educational resource management research focused on the analysis and discussion of the current state of educational resources, as well as recommendations for improving the current state of educational resources (Li and Sun, 2018). In addition, the research on the optimization of higher education management and the improvement of management strategies by technical methods is more detailed, and the research on the construction of an information platform in the recommendation for college educational resources is relatively lacking. The system of college education resource recommendation includes modules such as educational resource acquisition, educational resources, recommended resources, personal education resources, and subject information management. A T–S fuzzy neural network structure based on educational resource recommendation is constructed (Lu et al., 2020).

Feedforward neural network has a multilayer perceptron structure. The multilayer perceptron treats each input data as a vector $x=\left(x_{1},\;x_{2}\right)\text{,}$ and obtains the transfer function (Ozanich et al., 2020), as shown in Eq. 15:


(15) $$f\left(x\right)=x\cdot w+b$$

w is the weight vector, and b is the vertical offset (Gallab et al., 2019).

Nonlinear factors are introduced. The perceptron can approximate any nonlinear function, and the activation function activates the transfer function. Finally, the result is shown in Eq. 16:


(16) $$h\left(x\right)=\left\{\begin{array}{l} 1:\mathrm{if}\;f\left(x\right)=x\cdot w+b>0\\ 0:\;\mathrm{others} \end{array}\right\}$$

**Figure 4:**
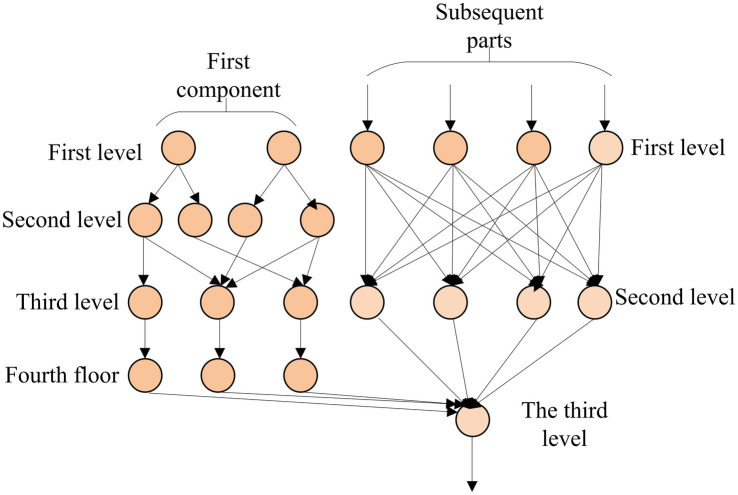
The structure of a multilayer perceptron.

Based on the theory of fuzzy generalized predictive control based on T–S model, a higher education management resource recommendation model based on T–S fuzzy neural network is constructed. The details are shown in Figure 4.

In Figure 4, the neural network consists of an input, an output, and multiple hidden layers. Every neuron is a perceptron. The neurons of the input layer serve as the input to the hidden layer. The hidden layer’s neurons are used as the input of the output layer, and the vector is input to the input layer. After repeated operations in the hidden layer, the output layer finally outputs the result of the neural network.

### 2.3. Recommendation process of higher education management resources based on T–S fuzzy neural network

**Figure 5:**
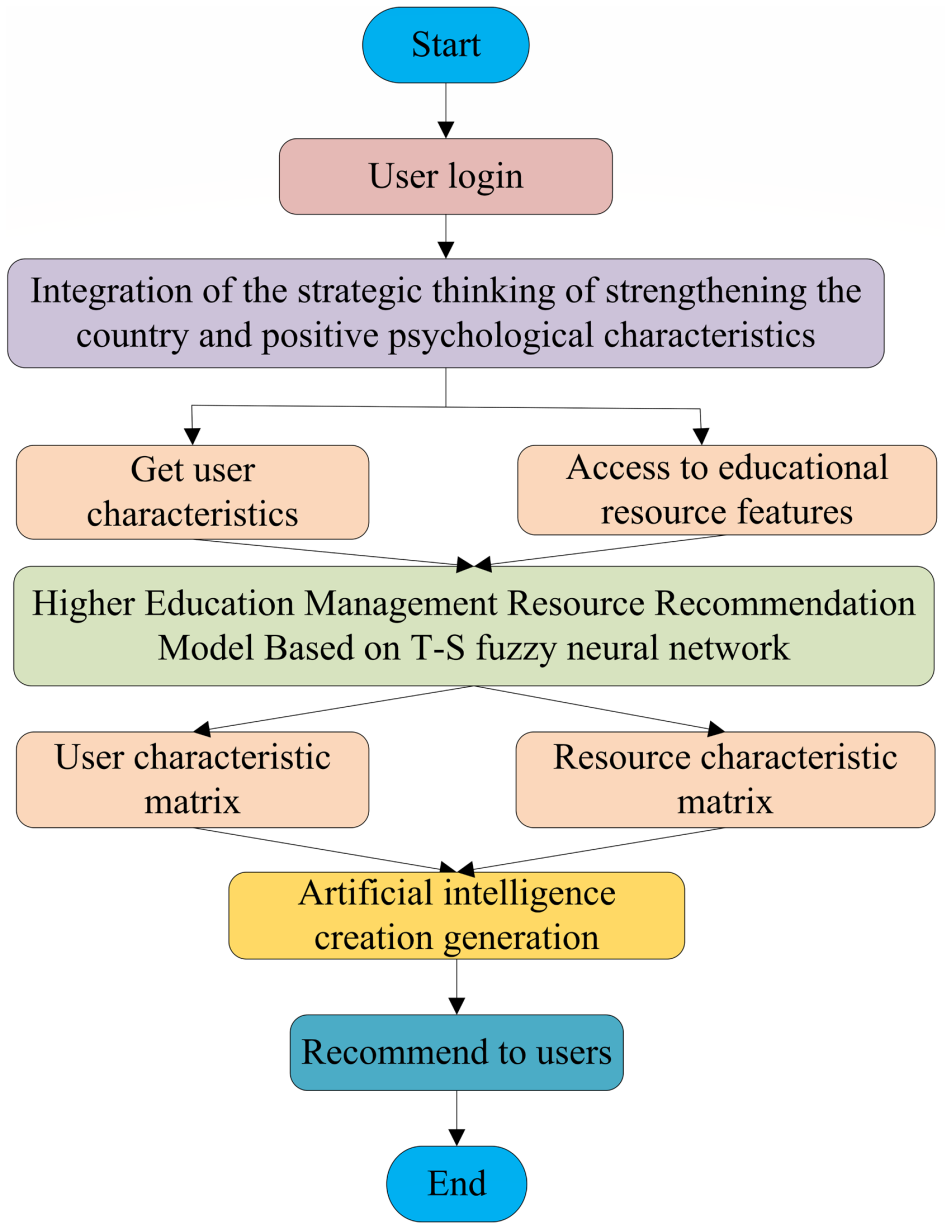
T–S fuzzy neural network educational resource recommendation model process.

This design is based on a neural network and fuzzy control algorithm, combined with the strategic thinking of strengthening the country through education, and under the action of positive psychology, a higher education management resource recommendation model based on a T–S fuzzy neural network is constructed, and the model process design is further carried out (Yao and Shekhar, 2021). The details are shown in Figure 5.

Figure 5 shows that starting from the user login, based on the strategic thinking of strengthening the country through education and the characteristics of positive psychology, through the data of user characteristics and educational resource characteristics, a T–S fuzzy neural network-based higher education management resource recommendation model is constructed. Further, a user feature matrix and an educational resource feature matrix are formed. The model recommends the most suitable resources to users through repeated calculations and predictions, and after multiple feedback corrections, and the entire recommendation process ends.

### 2.4. Investigation method and model demonstration

The first part is a simulation experiment of the educational resource recommendation design of the T–S fuzzy neural network. The second part is a questionnaire survey on the current situation of educational resources in colleges and universities and the application of educational resource recommendation design. The results before and after the design application are compared and analyzed.

#### 2.4.1. Experimental test

The system is tested by laboratory simulation, which verifies the feasibility of the fuzzy predictive control model based on neural networks in the recommendation of educational resources. Test requirements: the recommended educational resources must conform to the characteristics of users. That is, they must be related to the actual needs of users, and the recommendation accuracy rate should be >85%. In the experiment, the model is first trained and evaluated, and then tested and evaluated. Among them, the training evaluation time is 2 h, the training times of the model are 1,000 times, and the convergence time of the model is 1 h.

The test environment is shown in Table 1.

**Table 1:** Settings for the test environment.

Configure	Information
Laptop	Dell Inspiron 3,420
Operating system	Window10 64
Processor	Intel(R) Core (TM) i5-321OM
Memory	8G
Database	MySQL Server 5.6

#### 2.4.2. Design part of the questionnaire

Through the method of a questionnaire, taking the students of M colleges and universities in Xinjiang in the western region as research objects, the basic situation of students’ learning and teaching, and the effect of teaching resource recommendation design and application are investigated. The current situation of teaching resources, the influence of positive psychology on teaching effects, and the teaching situation before and after the application of the educational resource recommendation model for higher education management are investigated. In order to ensure the authenticity and reliability of the questionnaire content, the validation of the questionnaire is tested. Six education experts are invited to give a comprehensive evaluation of the rationality of the questionnaire content design. The specific results are shown in Table 2.

**Table 2:** Statistical table for questionnaire validity test.

Validity	Expert 1	Expert 2	Expert 3	Expert 4	Expert 5	Expert 6	frequency	percent (%)
Very reasonable	√		√	√		√	4	66.67
More reasonable		√			√		2	33.33
Not very reasonable							0	0
Unreasonable							0	0

The second part is the distribution and collection of questionnaires. At M universities, a questionnaire survey was administered to freshmen, sophomores, juniors, and some teachers. This survey adopts a small program of asking questions and using paper questionnaires. A total of 300 questionnaires were distributed, 290 were collected, and 280 were valid, for an effective rate of 96.6%. Statistical Product Service Solutions (SPSS) 22.0 was used to sort out, count, and analyze the questionnaires. Among them, 66.67% of the questionnaires are very reasonable, and 33.33% are relatively reasonable. In this paper, the reliability and validity of the questionnaire are tested, and the reliability coefficient of the questionnaire is 0.860 and the validity coefficient is 0.710. The results show that the questionnaire design is reasonable.

## 3. Test results and investigation analysis

### 3.1. Analysis of the current situation of the investigation of educational management resources in colleges and universities

#### 3.1.1. Analysis of the current situation of teaching faculty

**Figure 6:**
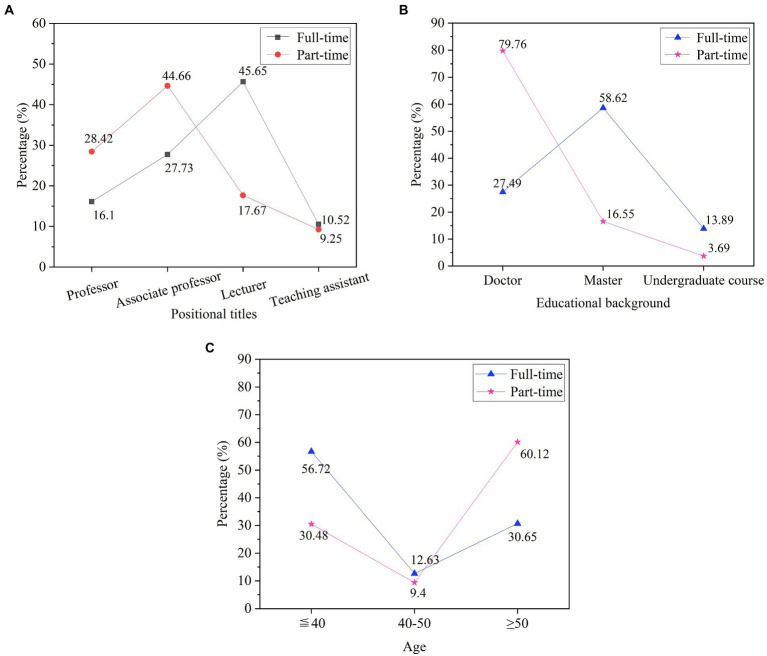
Current status of teaching faculty. **(A)** the professional titles; **(B)** the academic qualifications; **(C)** the age.

In order to understand the details of the teaching staff in detail, the three aspects of the teaching staff’s professional title, education background, and age are analyzed. All personnel are divided into full-time and part-time, as shown in Figure 6.

In Figure 6, full-time teachers account for the largest proportion of lecturers, accounting for 45.65%, followed by associate professors, accounting for 44.66%, professors, 16.1%, and teaching assistants, 10.52%. Among the part-time teachers, associate professors account for the largest proportion, accounting for 44.66%, followed by professors, accounting for 28.42%. The overall proportion of school professors is relatively low. In terms of academic qualifications, master’s degree is the main force among full-time teachers, accounting for 58.62%, followed by doctors, accounting for 27.49%, and some undergraduates, accounting for 13.89%. Among the part-time teachers, doctors are the most common, accounting for 79.76%. It shows that the academic qualifications of full-time teachers in the school are generally not high. From the perspective of age composition, most of them are under 40 years old, followed by those over 50 years old. There are not many full-time, young and experienced teachers. Full-time teachers are not strong enough.

#### 3.1.2. Analysis of the basic situation of school professional resources

**Figure 7:**
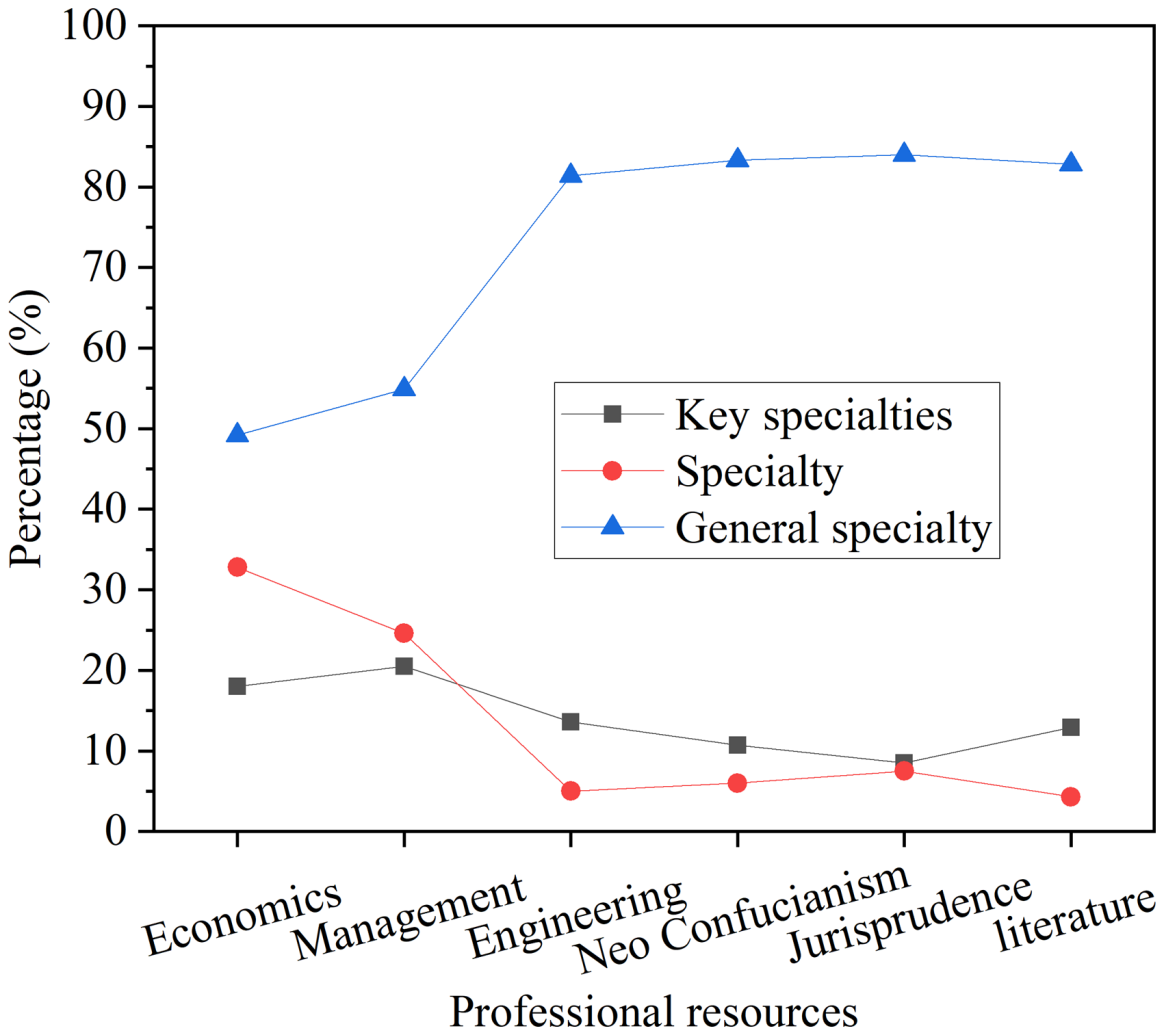
Analysis of professional resources in different disciplines.

The professional resources of the school are analyzed based on the proportion of key majors, characteristic majors, and general majors among the different disciplines of economics, management, engineering, science, law, and literature. The results are shown in Figure 7.

In Figure 7, in the situation of professional resources, the number of key majors is relatively small, the proportion of key majors and characteristic majors in economics and management disciplines is slightly higher, and other disciplines are generally lower. The school has a relatively large proportion of general majors. Professional advantages do not have great competitiveness, and the advantages are not obvious.

### 3.2. Feasibility analysis of application of higher education management resource recommendation model

**Figure 8:**
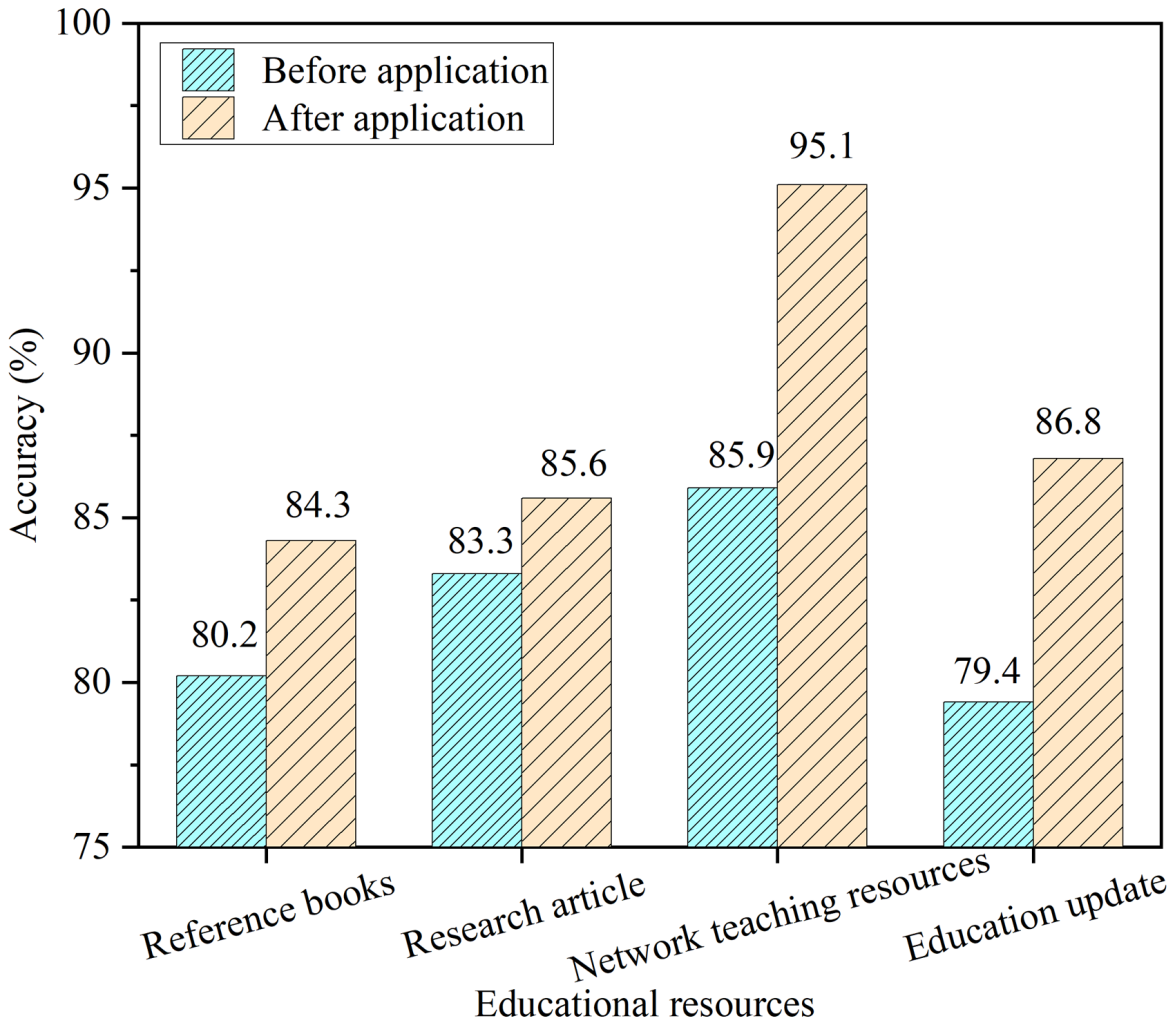
Comparison of educational resource recommendation results before and after model application.

According to the educational resource recommendation model, for the educational resources recommended by the system platform, including reference books, research articles, online teaching resources, and the latest educational information, the accuracy of educational resource recommendation is compared before and after, and the results are shown in Figure 8.

**Figure 9:**
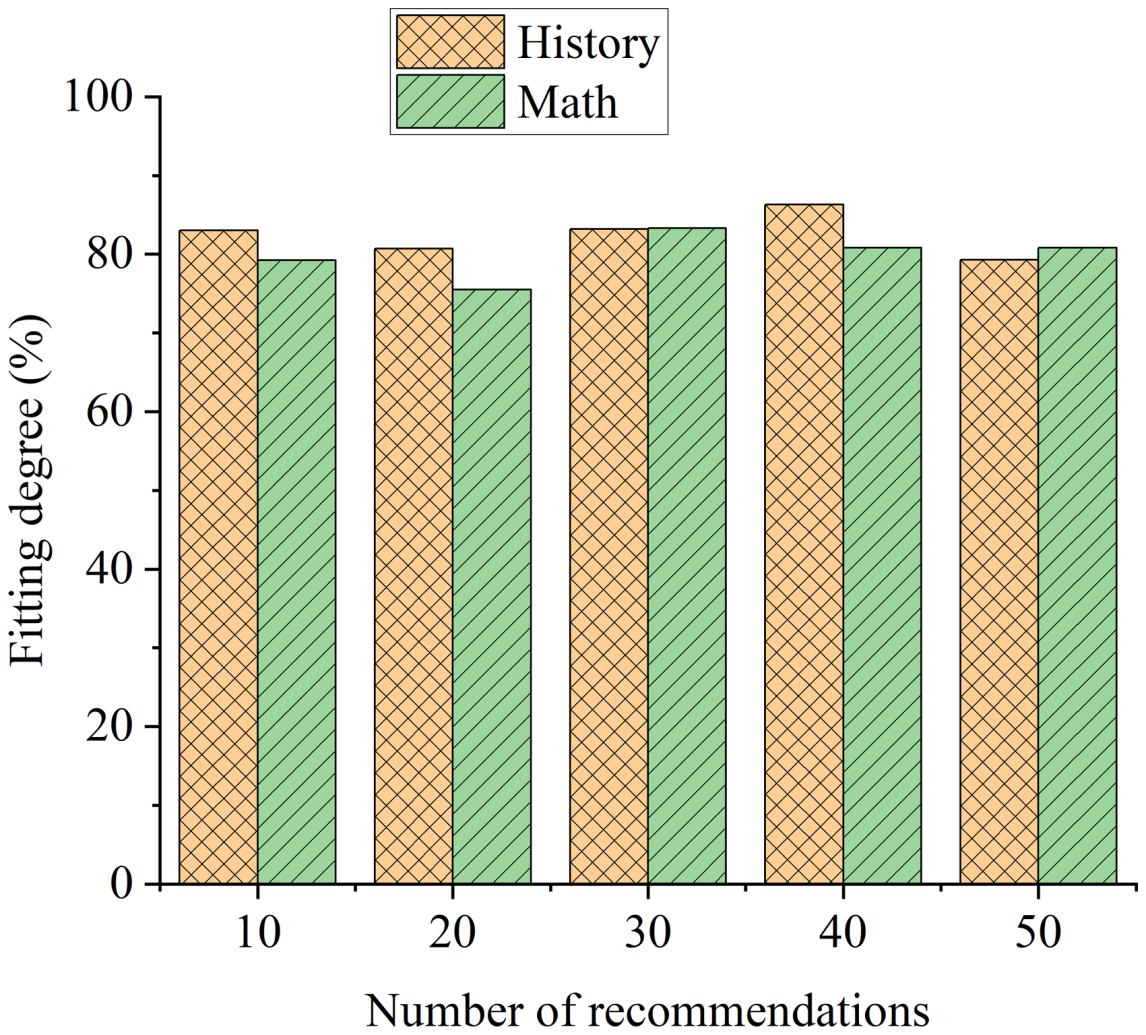
Evaluation of recommendation effect of model educational resources.

Figure 8 shows that after the application of this model, the recommendation accuracy of reference books, research papers, online teaching resources, and the latest educational information recommended by the platform has improved. The recommendation accuracy of reference books increased from 80.2% to 84.3%, that of research articles from 83.3% to 85.6%, that of online teaching resources from 85.9% to 95.1%, and that of the latest educational information from 79.4% to 86.8%. The accuracy rate has been improved on the whole. This shows that the design model is feasible. In addition, this paper also evaluates the effect of the model in the process of recommending actual educational resources, as shown in Figure 9, which shows the results of this paper’s evaluation of the effect of the model in recommending history and mathematics curriculum resources.

In Figure 9, the model designed in this paper performs very well in the research of curriculum resource recommendations in colleges and universities. The highest fitting degree of the model in the recommendation of teaching management resources for history and mathematics courses is about 83%, and the lowest is about 75%. It can be seen that the model designed in this paper has a very good effect on the recommendation of teaching management resources.

### 3.3. Analysis on the effect of higher education teaching management integrating positive psychological emotions

#### 3.3.1. The influence of positive psychological emotions on teachers’ work attitude

**Figure 10:**
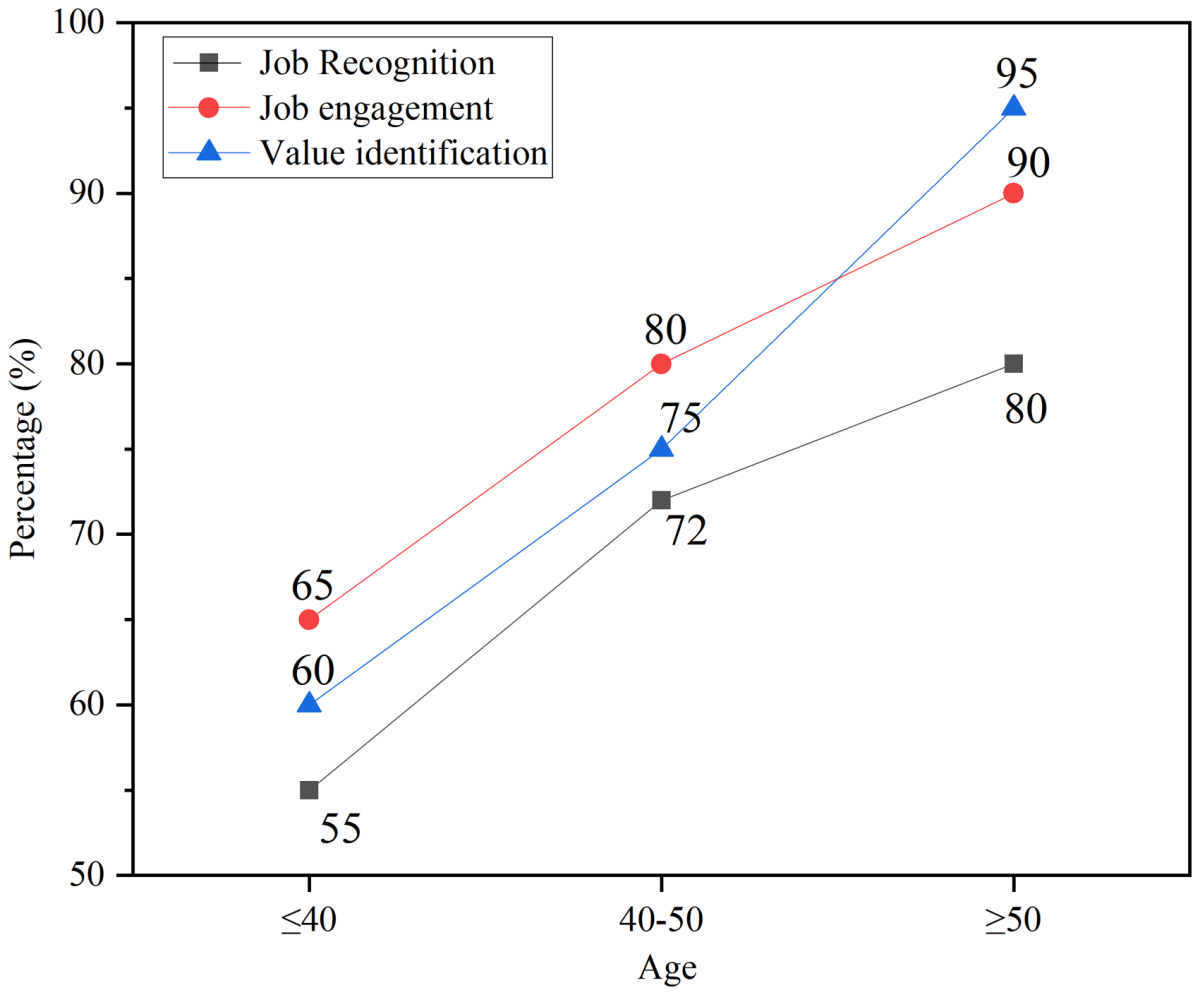
The influence of psychological emotions on teachers’ work attitude.

For teachers of different ages, the influence of positive psychological emotions on teachers’ work recognition, work engagement and value recognition in education and teaching management are analyzed, as shown in Figure 10.

In Figure 10, older teachers have higher recognition of their own work and are more engaged in their work. The stronger the sense of value recognition of the work, the deeper the qualifications and the longer the working hours, the more contributions to the society, the more positive emotions affect them, and the more positive work attitudes are promoted. Young people need to have a more positive attitude toward work to meet new tasks and improve their awareness of the value of work.

#### 3.3.2. The influence of positive psychological emotions on teachers’ psychological state

**Figure 11:**
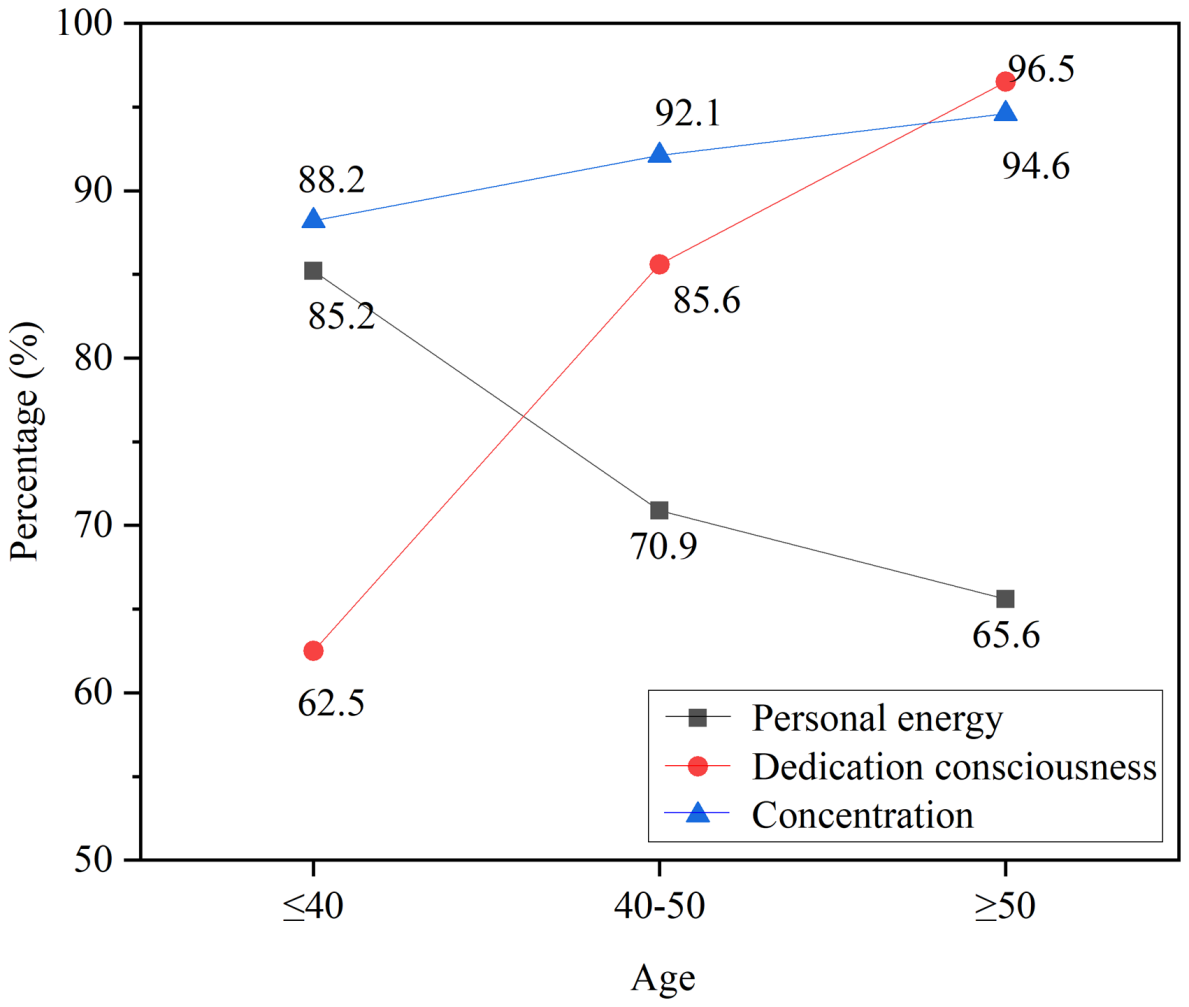
The influence of psychological emotions on teachers’ psychological state.

For teachers of different ages, the impact of positive psychological emotions on personal vitality, dedication, and concentration is analyzed, as shown in Figure 11.

In Figure 11, among teachers under the age of 40, the personal vitality is still relatively strong, and the vitality gradually decreases with age. In terms of dedication, teachers over the age of 50 have a stronger sense of commitment, followed by teachers aged 40–50, and finally, teachers under the age of 40. The gap in concentration is not very big, they can maintain good concentration, and teachers over 50 are slightly better. Positive psychological emotions can promote and enhance teachers’ dedication and concentration to a certain extent.

#### 3.3.3. The influence of positive psychological emotions on students’ conflict handling

**Figure 12:**
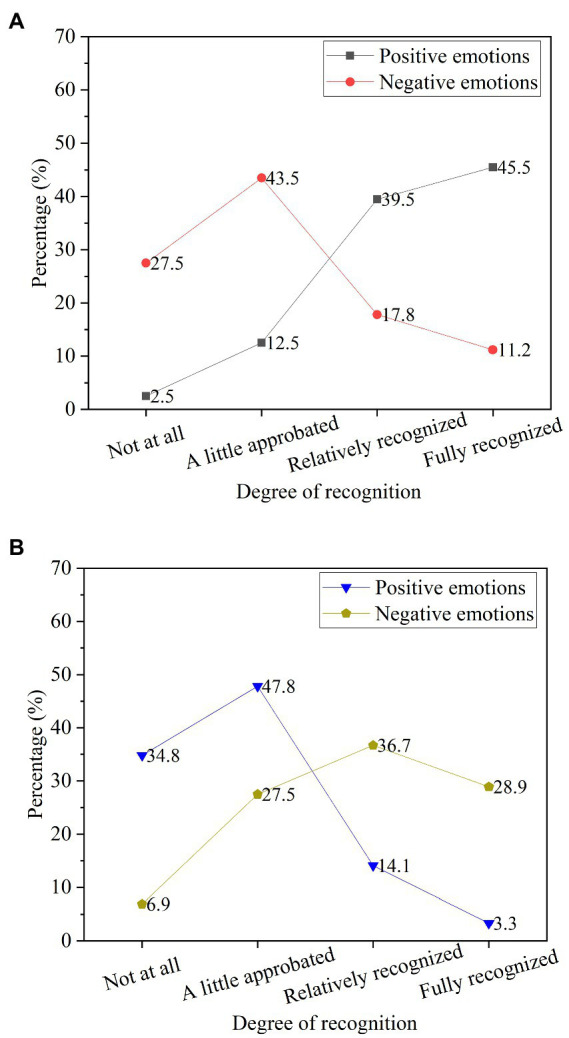
The influence of psychological emotions on the problem solving of college students. **(A)** The recognition of different psychological emotions for calmly analyzing and resolving conflicts; **(B)** The recognition of different psychological emotions for intensifying conflicts and behavioral confrontation.

The interpersonal aspect is mainly manifested in the communication and conflict resolution among the classmates. Rational resolution and calm analysis of problems and contradictions among college students under psychological and emotional conditions and recognition of behaviors and confrontations caused by intensified conflicts are analyzed, as shown in Figure 12.

In Figure 12, after conflicts arise among students, more students will calmly analyze and rationally resolve conflicts with positive psychological emotions. Under a negative psychological mood, the proportion of students’ ability to calmly analyze and rationally resolve conflicts decreases. A positive psychological state has a positive effect on conflict resolution. Positive psychological emotions can reduce the possibility of intensifying conflict and the possibility of behavioral confrontation.

### 3.4. Analysis of the impact of the application of the teaching resource recommendation model on the teaching effect

#### 3.4.1. Satisfaction analysis of query results of teaching resources under model application

**Figure 13:**
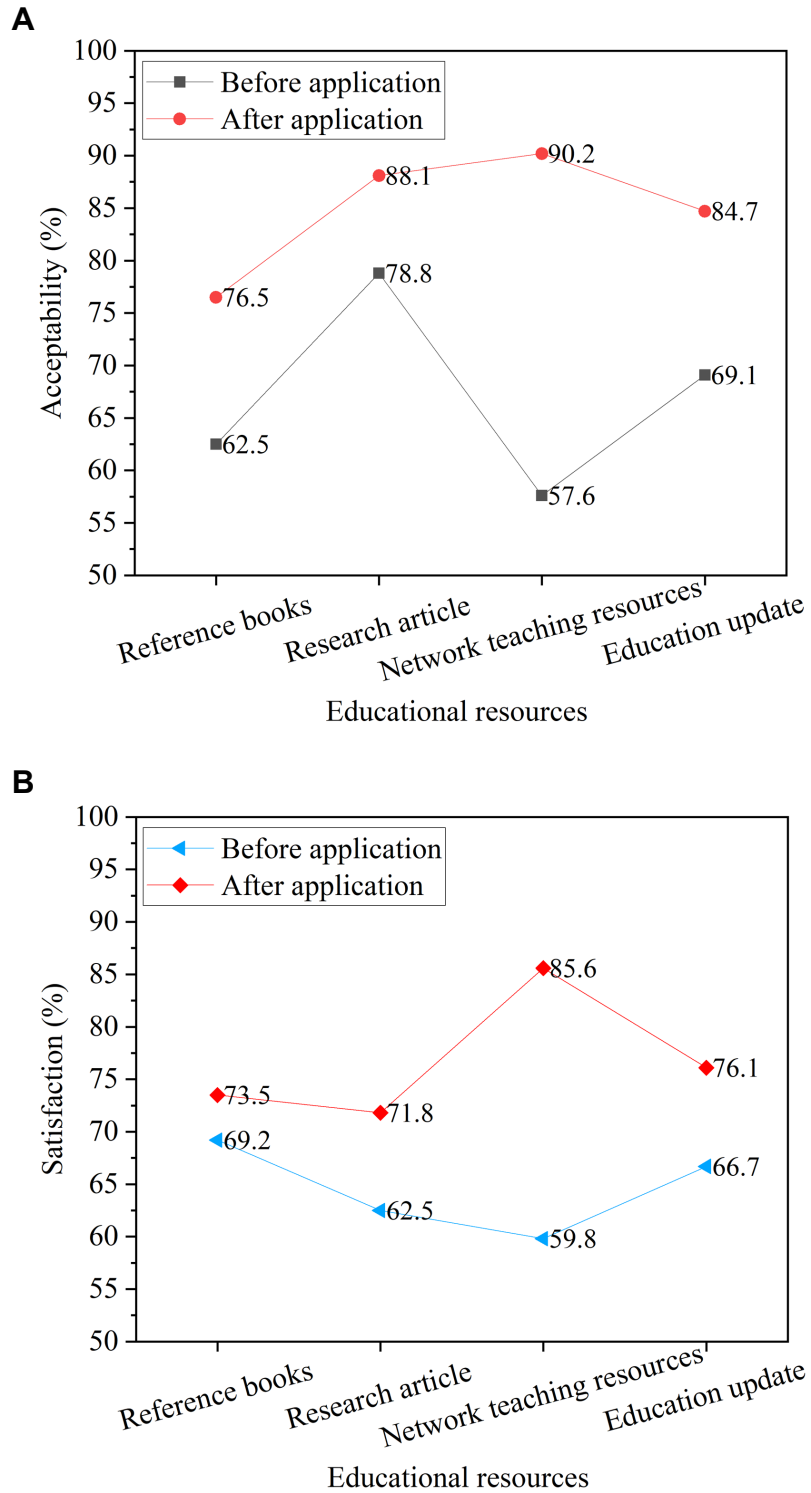
Satisfaction of teaching resource query results. **(A)** Comparison of acceptance of teaching resource query results; **(B)** Satisfaction comparison of teaching resource query efficiency.

Comparing the acceptance of the teaching resource query results and the satisfaction of the query efficiency of college students before and after the application of the teaching resource recommendation model, the results are shown in Figure 13.

Figure 13 shows that after the design and application of the recommended reference books, research articles, online teaching resources and education latest information, the acceptance of the query results and the satisfaction of query efficiency of the four types of resource recommendation have improved, especially for the query results of online teaching resources. The acceptance rate is the strongest, with the proportion increasing from 57.6% to 90.2%. In terms of query efficiency, satisfaction with the query efficiency of online teaching resources is the highest, ranging from 59.8% to 85.6%. Overall, college students are satisfied with the application of the teaching resource recommendation model in resource query.

#### 3.4.2. Satisfaction analysis of teaching resource application under model application

**Figure 14:**
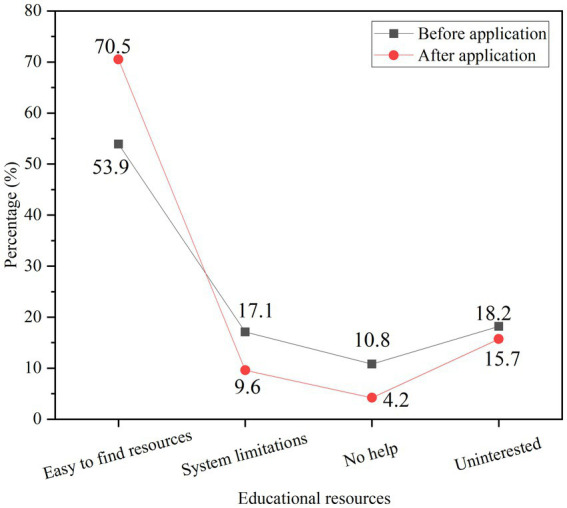
Comparison of teaching resource application satisfaction.

The degree of satisfaction of college students with the application of teaching resources before and after the recommended design and application of teaching resources is compared and analyzed, and the results are shown in Figure 14.

Figure 14 shows that after the application of the teaching resource recommendation model, the proportion of resource search convenience has increased, from 53.9% to 70.5%. The percentage of people who thought it was not helpful after the application dropped, from 10.8% to 4.2%. The percentage of people who felt the system had limitations fell from 17.1% to 9.6%. The teaching resource recommendation model can enhance students’ interest in the application of teaching resources as a whole, and application satisfaction is significantly improved.

## 4. Conclusion

In order to improve the level of higher education management in the western region, this study first conducts an in-depth study on the strategic thought of strengthening the country through education and the impact of positive psychology on higher education management, and then uses the T–S fuzzy neural network model to construct an educational resource recommendation based on the T–S fuzzy neural network. The model is designed, the process design is carried out, and the model is verified and tested. A questionnaire survey is used to conduct a comprehensive analysis of the current state of educational resources in colleges and universities, as well as the application effect of the resource recommendation model. The results show that: (1) When analyzing the current situation of the M College Education Resources Survey, the overall education of full-time teachers is not high, and the proportion of young full-time teachers with certain experience is relatively small. The school has a large proportion of ordinary majors, and the professional advantages are not competitive and not obvious. (2) After the model is applied, the recommendation accuracy of the four types of educational resources has improved, and the design is feasible. (3) Positive psychological emotions have a significant impact on the effectiveness of higher education management teaching. Positive psychological emotions can greatly enhance teachers’ sense of dedication and concentration, and positive psychological emotions can reduce the possibility of intensifying conflicts and behavioral confrontation. (4) College students are generally satisfied with the teaching resource recommendation model. The teaching resource recommendation model can enhance college students’ interest in the application of teaching resources, and application satisfaction is significantly improved. The limitation of this research is that, due to limited ability, the scope of the survey object is not broad enough, and future research should broaden the scope of the research and conduct experimental and popularized research in other universities in the western region. The main contribution is to use the relevant theories of positive psychology to construct the recommendation of higher education management resources based on T–S fuzzy neural networks in the western region, to investigate and analyze the teaching status and the feasibility and effect of the model application, and to improve the research on the recommendation system of educational resources in educational management, which improves the recommendation effect of educational resources.

## Data availability statement

The original contributions presented in the study are included in the article/supplementary material, further inquiries can be directed to the corresponding author.

## Author contributions

XMS contributed to conception and design of the study, organized the database and performed the statistical analysis, wrote the first draft of the manuscript, and wrote sections of the manuscript. XMS contributed to the manuscript revision, read, and approved the submitted version.

## Funding

This work was supported by the High-level Talents Scientific Research Initiation Project of Shihezi University (no. RCSK202001), Shihezi University Youth Innovation and Cultivation Talent Project (no. CXPYSK202103), and Independent Financial Support Project of Shihezi University (no. ZZZC202168).

## Conflict of interest

The author declares that the research was conducted in the absence of any commercial or financial relationships that could be construed as a potential conflict of interest.

## Publisher’s note

All claims expressed in this article are solely those of the authors and do not necessarily represent those of their affiliated organizations, or those of the publisher, the editors and the reviewers. Any product that may be evaluated in this article, or claim that may be made by its manufacturer, is not guaranteed or endorsed by the publisher.
